# Addressing Fecal Incontinence in Inflammatory Bowel Disease Patients: A Messy Problem

**DOI:** 10.1093/crocol/otab014

**Published:** 2021-02-24

**Authors:** Kofi Clarke

**Affiliations:** Penn State College of Medicine, Milton S Hershey Medical Center, Hershey, Pennsylvania, USA

Fecal incontinence (FI) is the involuntary passage of or inability to control expulsion of solid or liquid feces. It affects approximately 2%–10% of adults in the general population, can be socially embarrassing, and result in significant challenges with personal hygiene.^1^

Other studies estimate prevalence of FI in noninstitutionalized US adults at 8.3% (95% confidence interval: 7.1–9.5) with a breakdown of stool consistency as liquid stool in 6.2%, solid stool in 1.6%, and mucus in 3.1%.^2^

Specific to inflammatory bowel disease (IBD) patients, data on FI in varies widely in estimated prevalence and predisposing factors. This is likely due the methodology of various studies, as well as patients and providers not discussing or documenting FI as part of clinical visits.

A recent large study, Dibley et al. evaluated the effectiveness of asking about FI face to face and compared that to a brief self-completed survey in a cohort of IBD patients. FI was reported in 63% and 56%, respectively (*P* = 0.012) indicating the effectiveness of both methods. In addition, their results indicated that patients aged 51–60, with Crohn disease and higher disease activity, were more likely to report FI. Significantly 38.7% of respondents were interested in receiving help for their incontinence.^3^

In a retrospective study using the well phenotyped, large IBD SPARC registry, Kamal et al. reported FI in 1 in 6 IBD patients in *this issue of CC360*. Their results indicate that patients with ulcerative colitis and Crohn disease are equally affected. Older age, disease duration >15 years, and increased disease activity were noted to be significant risk factors as opposed to gender, disease location, and phenotype, as well as perianal disease which do not appear to be risk factors for FI.

The systematic review by D’Amico et al. which included 22 studies that enrolled IBD patients provides some guidance on evaluation tools that could be consistently adopted for evaluation of FI in IBD patients. This could help provide some consistency clinically and in research studies. They concluded that perhaps the Wexner score be used as the first step for assessment in IBD trials with anal manometry and/or endoanal ultrasonography used as follow-up in the case of positive questionnaires.^4^

Various treatment modalities with varying degrees of success have been evaluated across several studies. These include surgery, sacral nerve stimulation, and percutaneous tibial nerve stimulation. Gut directed behavioral treatment has proven to be quite effective and also improves functional gut symptoms.^5,6^ Furthermore, Zhang et al. in the study evaluating a subgroup in the NHANES 2009–2010 cohort noted that zinc intake was independently associated with increased risk of bowel leakage of gas, mucus, and liquid.^7^

Results from various studies with somewhat discordant findings emphasize the need to find common ground and answers to this messy problem:

1. Larger multicenter studies with simple predefined evaluation tools to better understand the scope and subgroup specific issues with FI in IBD patients are needed.
2. For research purposes, consistency of clinical practice and comparing various studies, investigators and clinicians should consider adoption of standardized scoring evaluation tools.
3. Finally, treatment options including complementary modalities need further evaluation for effectiveness and adoption to help our patients.

The recognition and better understanding of FI in IBD patients is a beginning, further collaborative research is needed to help us improve the quality of life for our patients with FI.

## Conflict of Interest

Speakers Bureau—Pfizer, Takeda, Janssen, and ABBVie. Research grant review—Pfizer.
